# Aberrant activation of IL-6/JAK/STAT3/FOSL1 signaling induces renal abnormalities in a *Xenopus* model of Joubert syndrome-related disorders

**DOI:** 10.1016/j.jbc.2025.110413

**Published:** 2025-06-24

**Authors:** Udval Uuganbayar, Hiromasa Ninomiya, Issei S. Shimada, Chisato Yamada, Mayu Kanie, Shinji Kawai, Takahiro Asai, Toru Miyoshi-Akiyama, Masayuki Itoh, Yutaka Hashimoto, Yoichi Kato

**Affiliations:** 1 Department of Cell Biology, Graduate School of Medical Sciences, Nagoya City University, Nagoya, Japan; 2 Department of Infection Diseases, National Center for Global Health and Medicine, Tokyo, Japan; 3 Department of Molecular and Cellular Biology, National Center of Neurology and Psychiatry, Kodaira, Japan

**Keywords:** cilia, Joubert syndrome related disorders, nephronophthisis, CEP290, IL-6, FOSL1, *Xenopus*

## Abstract

*CEP290* gene mutations are linked to Joubert syndrome-related disorders (JSRD) which present with various symptoms, including brain malformation, retinal degeneration, and kidney disorders. It remains unclear how patients with JSRD having *CEP290* gene mutations lead to kidney disorders, particularly polycystic kidney disease including nephronophthisis (NPH). To address this question, *Xenopus* CEP290 (xCEP290) was depleted using morpholino oligonucleotides against *xCEP290* in *Xenopus* embryos. xCEP290 morphants exhibited edema and dilated pronephric tubule, indicative of renal dysfunction. Next, RNA-seq analysis was performed to explore which signals and molecules are important for the formation of dilated pronephric tubule observed in the xCEP290 morphant kidney. The hallmark gene set associated with the IL-6/JAK/STAT3 signaling pathway was upregulated in xCEP290 morphant kidney, and inhibition of this signaling by JAK inhibitor ruxolitinib suppressed the dilated pronephric tubule in xCEP290 morphants. Furthermore, the expression level of transcription factor *Xenopus FOSL1* (*xFOSL1*), whose gene expression is regulated by IL-6 signaling, was upregulated in xCEP290 morphant kidney, and overexpression of xFOSL1 induced pronephric tubular dilation. These results together revealed that abnormal activation of IL-6/JAK/STAT3/FOSL1 signal axis is responsible for dilated pronephric tubule resembling cystic lesions observed in polycystic kidney disease of JSRD patients with *CEP290* gene mutations.

Primary cilia are antenna-like organelles on the surface of almost all cells in the human body, including renal tubular epithelial cells, and function as a sensor for external chemical or mechanical stimuli. In particular, cilia on the renal tubular epithelial cells detect fluid dynamics and transduce external stimuli into the intercellular signals, thereby preserving renal tubular integrity and maintaining homeostasis (1, 2, 3, 4, 5, 6, 7, 8). Defects in ciliary structure or function cause developmental disorders known as ciliopathies including Joubert syndrome-related disorders (JSRD). Pathogenic mutations in genes responsible for ciliopathies produce systemic symptoms such as brain malformation, retinal degeneration, and kidney abnormalities (9, 10). JSRD is characterized by malformation of cerebellar vermis called as “molar tooth sign” (11, 12), and approximately 30% of patients with JSRD have life-threatening renal dysfunction including nephronophthisis (NPH) characterized by renal cysts, interstitial cell infiltration as well as fibrosis (13, 14). Currently, no specific treatment for kidney disorders including NPH has been established, but renal dialysis or kidney transplantation may be necessary for end-stage renal failure when kidney disorders progress (9, 15). The *CEP290* gene has been reported as one of the causative genes for patients with JSRD (16, 17, 18, 19), and *CEP290* mutations are linked to the development of NPH (14, 20). Human *CEP290* gene, also known as *NPHP6*, is located on chromosome 12 and comprises 54 exons encoding the 290 kDa nephrocystin-6 protein composed of 2476 amino acids (21). This protein is localized to not only the ciliary transition zone in the cell that functions as a gatekeeper for translocating molecules in and out of cilium (22, 23, 24) but also to the centrosome and nucleus (21). However, how mutations in the CEP290 (nephrocystin-6) protein regulate the pathogenesis of JSRD remains poorly resolved.

Interleukin-6 (IL-6) is a pleiotropic cytokine that plays a central role in immune response and influences a wide array of physiological and pathological processes, including inflammation, cancer as well as autoimmune disease (25, 26). IL-6 signaling is transduced through two pathways, a classic signaling *via* membrane-bound IL-6 receptor (mIL-6R) and a trans-signaling mediated by the soluble IL-6R (sIL-6R) (26, 27, 28). Upon binding mIL-6R with IL-6, IL-6 induces dimerization of the signal-transducing glycoprotein 130 (gp130), activating a classic pathway, which involves the phosphorylation of Janus kinases (JAKs) that subsequently activates signal transducer and activator of transcription 3 (STAT3) (29). Phosphorylated STAT3 translocates to the nucleus and modulates the transcription of target genes leading to immune responses (30, 31, 32). IL-6 signaling has also been shown to play important roles in the pathogenesis of various kidney disorders, including chronic kidney disease (CKD), diabetic nephropathy (DN), acute kidney injury (AKI), and autosomal dominant polycystic kidney disease (ADPKD) (28, 33, 34, 35, 36, 37). Elevated IL-6 levels are further implicated in promoting hypertension and renal fibrosis in CKD, with angiotensin II serving as a potent inducer of IL-6 production (38). In patients with DN, IL-6 signaling predominantly activates gp130-STAT3-dependent signaling that produces inflammation, immune cell infiltration, and extracellular matrix remodeling (39). IL-6 signaling *via* STAT3 is further linked to the pathogenesis of ADPKD, since the expression level of IL-6 gene and protein is increased in patients with ADPKD (36, 37). While polycystic kidney disease is also accompanied with JSRD caused by ciliary abnormalities like ADPKD (40, 41, 42, 43, 44), the role of IL-6 signaling pathway in kidney disorders observed in JSRD has not yet been elucidated.

The FOS-like 1 (FOSL1)/FOS-related antigen-1 (Fra1), which belongs to the FOS family, is a canonical component of the activator protein-1 (AP-1) transcription factor (45, 46, 47). The FOSL1 is mainly activated by ERK1/2 and p38 MAPK signaling and interacts with JUN proteins to form the canonical AP-1 transcriptional complex (46). The transcription of *FOSL1* gene is directly regulated by STAT3 activated in response to IL-6 stimulation (48, 49, 50). FOSL1 is essential for various biological processes and pathologies such as proliferation, differentiation, survival, cell fate, stem cell reprogramming, epithelial–mesenchymal transition, inflammation, and tumorigenesis (46, 51, 52, 53). While abnormal expression of *FOSL1* gene has been implicated in renal pathologies such as renal cell carcinoma and AKI (54, 55), there is no report on the association between FOSL1 and polycystic kidney diseases so far.

Here we show tolvaptan, a vasopressin V2 receptor antagonist, which is prescribed for patients with ADPKD to slow renal cyst progression (15, 56), can suppress the formation of dilated pronephric tubules observed in xCEP290-depleted kidneys of *Xenopus* embryos. Furthermore, RNA-seq analysis using xCEP290-depleted kidneys with or without the tolvaptan treatment identified the hallmark gene set associated with the IL-6/JAK/STAT3 signaling pathway as a candidate signal that causes the formation of dilated pronephric tubules. Indeed, ruxolitinib, an inhibitor of JAK1/2 that is a component of IL-6 signaling, rescued dilated pronephric tubules as well as the activation of xSTAT3 in the pronephric tubular epithelial cells observed in xCEP290 morphants. Lastly, the expression of *xFOSL1*, a target gene of IL-6 signaling, was upregulated in xCEP290-depleted kidneys, and overexpression of xFOSL1 produced dilation of pronephric tubules. The dilated pronephric tubule induced by the depletion of xCEP290 was further rescued by the inhibition of xFOSL1 function. These results together strongly indicated that the activation of IL-6/JAK/STAT3/FOSL1 signaling axis is essential for the dilation of the pronephric tubule in the kidney of xCEP290-depleted embryos.

## Results

### Pronephric tubule was dilated in xCEP290-depleted embryonic kidney

**Figure 1:**
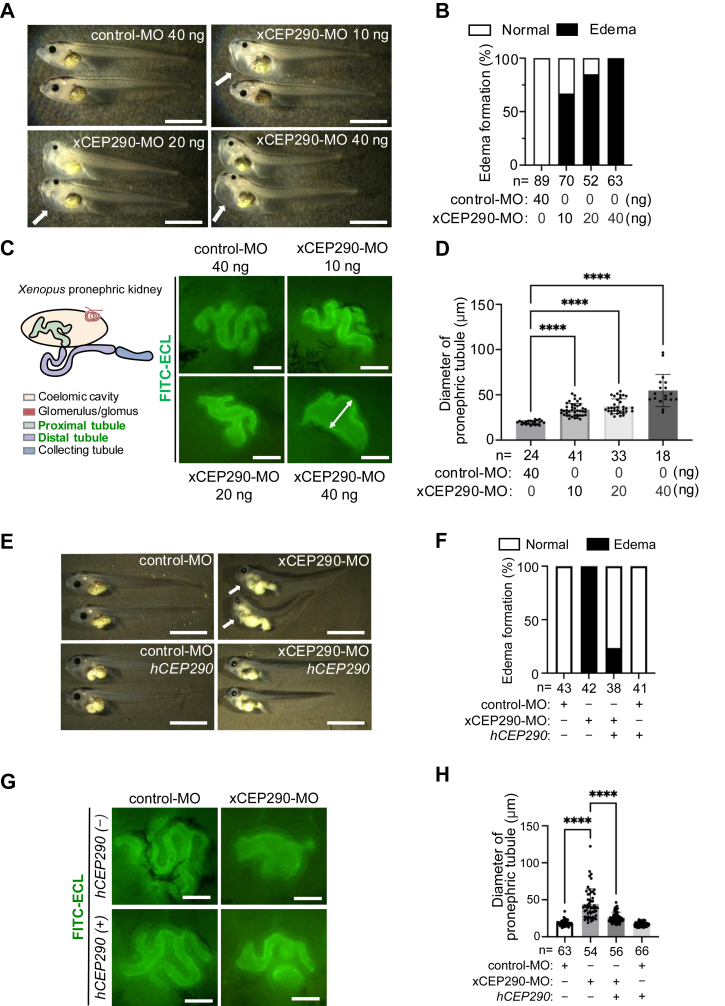
**xCEP290-depletion produced edema and dilated pronephric tubule in *Xenopus* embryo**. *A*, the edema formation observed in *x*CEP290 morphant. *Upper left panel*: control-MO (40 ng), *upper right panel*: xCEP290-MO (10 ng), *lower left panel*: xCEP290-MO (20 ng), and *lower right panel*: xCEP290-MO (40 ng). A typical edema in each *panel* is indicated by a *white arrow*. The scale bar represents 1 mm. *B*, the percentage of embryos with edema in examined embryos in (*A*). Each ‘n’ indicates the total number of embryos from three independent experiments. *C*, the schematic showing *Xenopus* pronephric tubular segmentated patterns (113). Each color represents coelomic cavity (*flesh color*), glomerulus/glomus (*red*), proximal tubule (*yellow green*), distal tubule (*purple*) and collecting tubule (*blue*). Proximal and distal pronephric tubules are stained by FITC-conjugated *Erythrina crista-galli* lectin (FITC-ECL: *green*) at stage 37. *Upper left panel*: control-MO (40 ng), *upper right panel*: xCEP290-MO (10 ng), *lower left panel*: xCEP290-MO (20 ng), and *lower right panel*: xCEP290-MO (40 ng). A typical expanded inner diameter of pronephric tubule is indicated by a *white arrow* in the *lower right panel*. The scale bar represents 50 μm. *D*, quantification of inner diameter in pronephric tubule of examined embryo in (*C*). Each ‘n’ indicates the total number of embryos from three independent experiments. ∗∗∗∗: *p* < 0.0001. *E*, edema formation in xCEP290 morphant with or without co-injection of *hCEP290* mRNA. *Upper left panel*: control-MO (40 ng), *upper right panel*: xCEP290-MO (40 ng), *lower left panel*: control-MO (40 ng) with *hCEP290* mRNA (500 pg), and *lowerright panel*: xCEP290-MO (40 ng) with *hCEP290* mRNA (500 pg). Edemas are indicated by *white arrows* in the *upper right panel*. The scale bar represents 1 mm. *F*, the percentage of embryos with edema in examined embryos in (*E*). Each ‘n’ indicates the total number of embryos from three independent experiments. *G*, pronephric tubule was visualized using FITC-ECL staining (*green*). *Upper left panel*: control-MO (40 ng), *upper right panel*: xCEP290-MO (40 ng), *lower left panel*: control-MO (40 ng) with *hCEP290* mRNA (500 pg), and *lower right panel*: xCEP290-MO (40 ng) with *hCEP290* mRNA (500 pg). The scale bar represents 50 μm. *H*, quantification of inner diameter in pronephric tubule of examined embryo in (*G*). Each ‘n’ indicates the total number of embryos from three independent experiments. ∗∗∗∗: *p* < 0.0001.

Previous studies have demonstrated that loss of CEP290 is linked to cilia-related kidney disorders as well as other phenotypes, including abnormalities of eyes, central nervous system, liver and heart (9, 16, 19, 57, 58, 59). To investigate the molecular mechanisms underlying the kidney disorders observed in JSRD with *CEP290* gene mutations, we depleted xCEP290 protein in *Xenopus laevis* embryos by injecting xCEP290 translational-blocking morpholino oligonucleotides (xCEP290-MO) (60). The protein expression level of the reporter construct xCEP290(-52–394)-2HA (xCEP290(-52–394)-2HA), which includes the target sequence of xCEP290-MO, was effectively abolished by xCEP290-MO co-injection in *Xenopus* embryos (Fig. S1*A*), demonstrating the effectiveness of the morpholino. xCEP290 morphants exhibited edema formation whereas no significant phenotype was observed in embryos with the injection of a control-MO (Fig. 1, *A* and *B*). This result indicates that xCEP290 deficiency in embryos may cause defects in circulating organ systems, including the kidney and heart (61). We first examined the morphology of pronephric tubule in xCEP290 morphant kidneys, which is subdivided into proximal and distal segments and the connecting tubule (62). The depletion of xCEP290 protein induced an elongated inner diameter of pronephric tubule when compared with control-MO injected embryos (Fig. 1, *C* and *D*). Rescue experiments were performed to show the specificity of these defects. The large size of the *CEP290* gene made it difficult to obtain the full-length clone and the full length of *Xenopus* CEP290 clone is not currently available. On the other hand, human *CEP290* (*hCEP290*), which does not include the target sequence of xCEP290-MO and whose protein sequence as well as the domain structures are conserved between human and *Xenopus* (The similarity between NP_079390.3 vs XP_031754760.1 is 63% examined by BLAST: https://blast.ncbi.nlm.nih.gov/Blast.cgi, and the domains were predicted by InterPro: https://www.ebi.ac.uk/interpro/and SMART: http://smart.embl-heidelberg.de/), was available, so we used the *hCEP290* construct for our rescue studies. Should rescue be observed, this would not only demonstrate functional conservation but evolutionary conservation. Indeed, the renal defects, edema and dilated pronephric tubule observed in xCEP290 morphants, were suppressed by co-injection of *hCEP290* mRNA (Fig. 1, *E*–*H*). We also assessed the length of primary cilia on the epithelial cells of pronephric tubules, since abnormal cilia on the renal tubular epithelial cells are observed in patients with JSRD and likely affect cyst formation, inflammation and fibrosis in the kidney (9, 63, 64, 65). xCEP290 morphants had significantly elongated cilia in the epithelial cells of pronephric tubules when compared to the control-MO injected embryos (Fig. S1, *B* and *C*). This ciliary phenotype was similar to that observed in patients with JSRD having *CEP290* gene mutations (19, 59, 66).

These findings show that xCEP290 deficiency in *Xenopus* embryos produce edema and dilated pronephric tubule accompanied with elongated primary cilia on the epithelial cells of pronephric tubule.

### The treatment of tolvaptan improved dilated pronephric tubule in xCEP290 morphant kidneys

**Figure 2:**
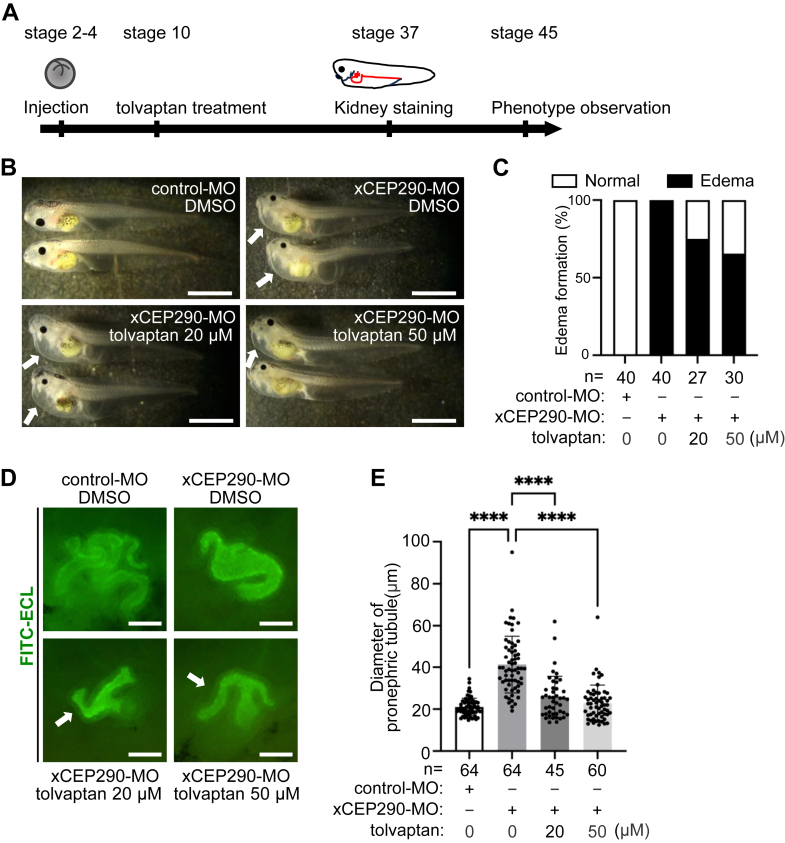
**The treatment of tolvaptan rescued renal phenotype in xCEP290 morphant**. *A*, schematic showing time scale of the tolvaptan treatment. control-MO (40 ng) and xCEP290-MO (40 ng) were injected into *Xenopus* embryo at stage 2 to 4; tolvaptan (50 μM) was treated from stage 10, followed by pronephric tubule staining at stage 37 embryo and observation of phenotype until stage 45. *B*, edema formation in xCEP290 morphant with the tolvaptan treatment. *Upper left* and *right panels*: control-MO (40 ng) and xCEP290-MO (40 ng) with DMSO treatment, respectively, *lower left* and *right panels*: xCEP290-MO (40 ng) with the tolvaptan treatment of 20 μM and 50 μM. Edema are indicated by *white arrows* in the *upper right panel* and *lower panels*. The scale bar represents 1 mm. *C*, the percentage of embryos with edema in the examined embryos in (B). Each ‘n’ indicates the total number of embryos from three independent experiments. *D*, pronephric tubule was visualized using FITC-ECL staining (*green*). *Upper left panel*: control-MO (40 ng), *upper right panel*: xCEP290-MO (40 ng) with DMSO treatment, *lower left panel*: xCEP290-MO (40 ng) with the tolvaptan treatment (20 μM), *lower right panel*: xCEP290-MO (40 ng) with the tolvaptan treatment (50 μM). Improved pronephric tubule is indicated by a *white arrow* in the *lower panels*, respectively. The scale bar represents 50 μm . *E*, quantification of pronephric tubular inner diameter in examined embryos in (*D*). Each ‘n’ indicates the total number of embryos from three independent experiments. ∗∗∗∗: *p* < 0.0001.

Currently, the drug tolvaptan is used to therapeutically slow the progression of renal cyst formation in patients with ADPKD (67, 68, 69, 70, 71, 72). We therefore examined the effect of tolvaptan on the edema and dilated pronephric tubule phenotype observed in the xCEP290 morphants. xCEP290 morphants were treated with different concentrations of tolvaptan at Stage 10 developing embryos, which is prior to renal development (Fig. 2*A*) and then observed until Stage 45 when the formation of edema is clearly confirmed (Fig. 2, *B* and *C*). The tolvaptan treatment decreased the formation of edema in xCEP290 morphants (Fig. 2*B*: lower panels, C).

Next, the morphology of the pronephric tubule in xCEP290 morphant kidney with the tolvaptan treatment was examined (Fig. 2, *D* and *E*). As we expected, the tolvaptan treatment shortened the elongated inner diameter of the pronephric tubule (Fig. 2*D*: lower panels, E), while neither 20 *μ*M nor 50 *μ*M tolvaptan treatment was able to restore the normal state of pronephric tubular extension in the comparison between normal and xCEP290-depleted embryos (Fig. 2*D*: upper left panel and lower panels). On the other hand, the elongated primary cilia on the tubular epithelial cells of xCEP290 morphants were not restored by the tolvaptan treatment (Fig. S2, *A* and *B*).

These results show that the tolvaptan treatment improved edema and dilated pronephric tubules but not cilia length observed in xCEP290 morphant.

### IL-6/JAK/STAT3 signaling is a candidate signaling responsible for the xCEP290 morphant phenotype

**Figure 3:**
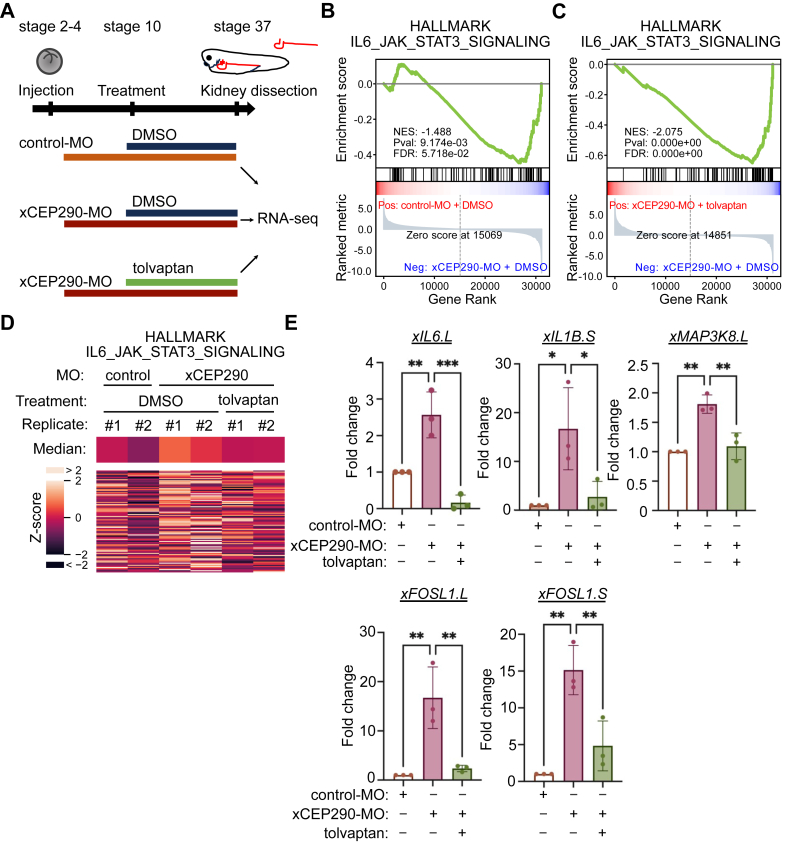
**Genes associated with IL-6/JAK/STAT3 signaling is up-regulated in xCEP290 morphant kidney**. *A*, experimental design of RNA-seq analysis. control-MO and xCEP290-MO (40 ng) were injected in *Xenopus* embryo at stage 2 to 4; DMSO and tolvaptan (50 μM) were treated from stage 10, followed by kidney dissection at stage 37. Fifty to sixty dissected kidneys were pooled for RNA-seq analysis. *B*, GSEA plot in HALLMARK_IL6_JAK_STAT3_SIGNALING between control-MO and xCEP290-MO in DMSO treatment condition. Normalized enrichment score (NES), *p*-values (Pval), and false discovery rate (FDR) are also shown. *C*, GSEA plot in HALLMARK_IL6_JAK_STAT3_SIGNALING between DMSO and tolvaptan treatments in xCEP290-MO condition. NES, Pval, and FDR are also shown. *D*, heatmap plot of genes in the HALLMARK_IL6_JAK_STAT3_SIGNALING gene set. The color of heatmap is the TPM value with z-score normalization. Heatmap of *middle row* indicates the median values of the gene set in the conditions and replicates, and that of *lower row* shows each gene in the gene set. *E*, validating the expression level of IL-6/JAK/STAT3 signaling related genes (*xIL6*.*L*, *xIL1B*.*S*, *xMAP3K8*.*L*, *xFOSL1*.*L*, and *xFOSL1*.*S*) in kidney by RT-qPCR. Expressions of genes were normalized with *xODC1* gene expression, and expressions in control groups were set as 1. Each data point represents the mean ± STDEV of three independent experiments were performed. ∗: *p* < 0.05, ∗∗: *p* < 0.01 and ∗∗∗: *p* < 0.001.

To explore the molecular mechanism of pronephric tubular dilation induced by xCEP290 knockdown, we took advantage of RNA-seq and multidimensional bioinformatics analyses. Since the tolvaptan treatment shortened the elongated inner diameter of the pronephric tubule in xCEP290 morphant kidney, we reasoned that genes, whose expression level is changed by xCEP290 knockdown and restored by the tolvaptan treatment, may be important for the formation of pronephric tubular dilation in xCEP290 morphant. RNAs were therefore extracted from dissected control or xCEP290 morphant kidneys with or without the tolvaptan treatment. RNAs from dissected control kidneys were examined by quantitative RT-PCR (RT-qPCR) assay with known kidney markers such as *xFXYD2* and *xPAX8* (73, 74) or other tissue markers such as *xACTC1* (muscle marker), *xNCAM1* (neural marker) and *xKRT12*.*4* (epidermis marker) (75, 76) to test for possible contamination from tissues other than the kidney (Fig. S3, *A*–*C*), and then RNA-seq was performed (Fig. 3*A*). We found that kidney samples from control embryos, as well as xCEP290 morphants with or without the tolvaptan treatment, revealed variations in global gene expression patterns displayed in the box plot (Fig. S4*A*). The distant genetic affiliation among three groups, which are control kidneys with DMSO and xCEP290 morphant kidneys with or without tolvaptan, was refined with the aid of principal component analysis (PCA) (Fig. S4*B*). To illuminate the “omics” features of pronephric tubular dilation, we turned to gene set enrichment analysis (GSEA) with HALLMARK gene sets and found the significantly different gene sets associated with IL-6_JAK_STAT3 signaling (*p* < 9.174e-3), MYC Targets V2 (*p* < 3.003e-3) and MYC Targets V1 (*p* < 1.241e-3) compared to control-MO with DMSO and xCEP290-MO with DMSO (Fig. 3*B*, Fig. S4, *C* and *D*). However, a gene set associated with IL-6_JAK_STAT3 signaling (*p* < 0.000) but not MYC Targets V2 and V1 was different when compared to xCEP290-MO with DMSO and tolvaptan (Fig. 3*C*, Fig. S4, *E* and *F*, Data S-1). The differentially expressed genes (DEGs) were enriched in IL-6_JAK_STAT3 signaling, MYC Targets V2, and MYC Targets V1, as shown by the heatmap diagram (Fig. 3*D*, Fig. S4, *G* and *H*). Some other signaling pathways were also modulated between control kidneys with DMSO and xCEP290 morphant kidneys with and without the tolvaptan treatment, were shown on Data S-1.

To further validate RNA-seq results, we selected genes associated with IL-6/JAK/STAT3 signaling and examined their expression in control kidneys with DMSO and xCEP290 morphant kidneys with or without the tolvaptan treatment by RT-qPCR. The expression of *xIL6*.*L*, *xIL1B*.*S*, *xMAP3K8*.*L*, *xFOSL1*.*L*, *xFOSL1*.*S*, *and xELF3*.*L* genes was examined (Fig. 3*E*, Fig. S4*I*). The expression of these genes was up-regulated in xCEP290 morphant kidney with DMSO when compared with the control kidney with DMSO, whereas they were down-regulated in xCEP290 morphant kidney with tolvaptan treatment when compared with xCEP290 morphant kidney with DMSO.

Collectively, these results uncover a gene set associated with IL-6/JAK/STAT3 signaling that is potentially involved in the formation of pronephric tubular dilation in xCEP290-depleted embryos.

### Inhibition of JAK activity recovered the dilated pronephric tubule in xCEP290 morphant

**Figure 4:**
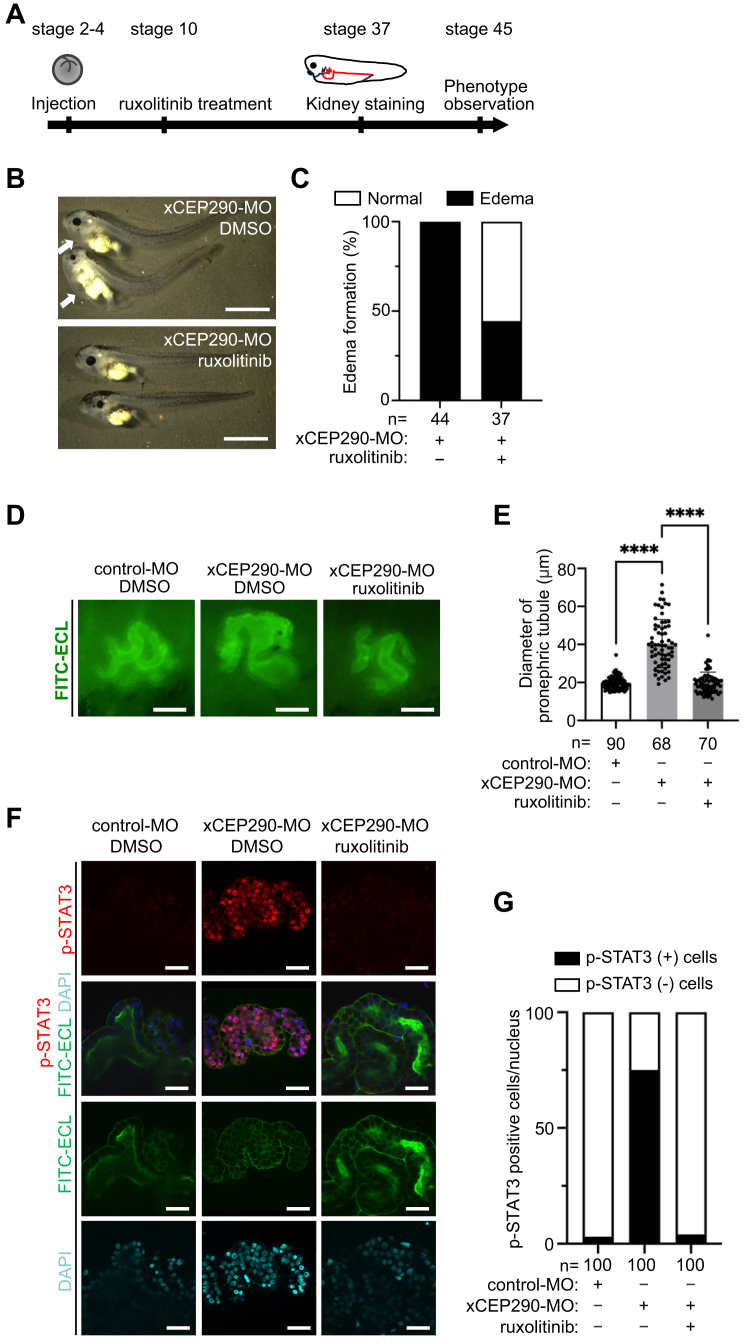
**Inhibition of the JAK activity rescued renal phenotype of xCEP290 morphant**. *A*, the schematic showing time scale of the ruxolitinib treatment. control-MO (40 ng) and xCEP290-MO (40 ng) were injected in *Xenopus* embryo at stage 2 to 4; ruxolitinib (1 μM) was treated from stage 10, followed by pronephric tubule staining at stage 37 embryo and observation of phenotype until stage 45. *B*, edema formation in xCEP290 morphant with or without the ruxolitinib treatment. *Upper panel*: xCEP290-MO (40 ng) with DMSO treatment, *lower panel*: xCEP290-MO (40 ng) with the ruxolitinib treatment of 1 μM. Edemas were indicated by *white arrows* in the *upper panel*. The scale bar represents 1 mm. *C*, the percentage of embryos with edema in examined embryos in (B). Each ‘n’ indicates the total number of embryos from three independent experiments. *D*, pronephric tubule was visualized using FITC-ECL staining (*green*). *left panel*: control-MO (40 ng), *middle panel*: xCEP290-MO (40 ng) with DMSO treatment, *right panel*: xCEP290-MO (40 ng) with the ruxolitinib treatment of 1 μM. The scale bar represents 50 μm. *E*, quantification of pronephric tubular inner diameter in examined embryos in (*E*). Each ‘n’ indicates the total number of embryos from three independent experiments. ∗∗∗∗: *p* < 0.0001. *F*, the number of p-STAT3 positive (*red*) pronephric tubular epithelial cells was increased in xCEP290 morphant kidney. Pronephric tubule and nucleus were visualized using FITC-ECL staining (*green*) and DAPI staining (*cyan*), respectively. The scale bar represents 20 μm. *G*, the percentage of p-STAT3 positive pronephric tubular epithelial cells in examined embryos in (*F*). Each ‘n’ indicates the total number of pronephric tubular epithelial cells from three independent experiments.

To determine whether the activation of IL-6/JAK/STAT3 signaling induces dilated pronephric tubules in xCEP290-depleted kidneys, the activity of JAK was blocked by ruxolitinib, a JAK1/2 inhibitor (77, 78). When xCEP290 morphants were treated with 1 μM ruxolitinib at stage 10, the number of xCEP290 morphants with edema was reduced (Fig. 4, *A* and *B*, *C*). Furthermore, the ruxolitinib treatment significantly shortened the elongated inner diameter of the pronephric tubule in xCEP290-depleted kidneys (Fig. 4, *D* and *E*), while the elongated primary cilia on the tubular epithelial cells of xCEP290 morphants were not restored by the ruxolitinib treatment (Fig. S5, *A* and *B*). The state of xSTAT3 activation was next examined in xCEP290 morphant kidney by immunohistochemistry using anti-phosphorylated-xSTAT3 (p-STAT3: Tyr705) antibody (79). The number of p-STAT3 positive pronephric tubular epithelial cells was abnormally increased in the xCEP290 morphant kidney compared with the control-MO injected kidney, and this increase was reduced to a normal level with the ruxolitinib treatment (Fig. 4*F*: top panels, G).

We also tested the involvement of other genes associated with IL-6/JAK/STAT3 signaling identified by RNA-seq analysis in the formation of dilated pronephric tubule (Fig. 3*E*, Fig. S4*I*). The effect of Tpl2 Kinase Inhibitor, an inhibitor for MAP3K8 (80) and auranofin, an inhibitor of protein kinase C iota (PKCί) that is upstream of ELF3 (81) was examined on the improvement of pronephric tubular dilation in xCEP290 morphants (Fig. S5, *C* and *D*). All inhibitors were observed to shorten the elongated inner diameter of the pronephric tubule in xCEP290-depleted kidneys.

These results strongly indicate that the activation of IL-6/JAK/STAT3 signaling causes the phenotype of xCEP290 morphant.

### Transcription factor FOSL1 functions downstream of IL-6/JAK/STAT3 signaling for dilated pronephric tubule in xCEP290 morphant

**Figure 5:**
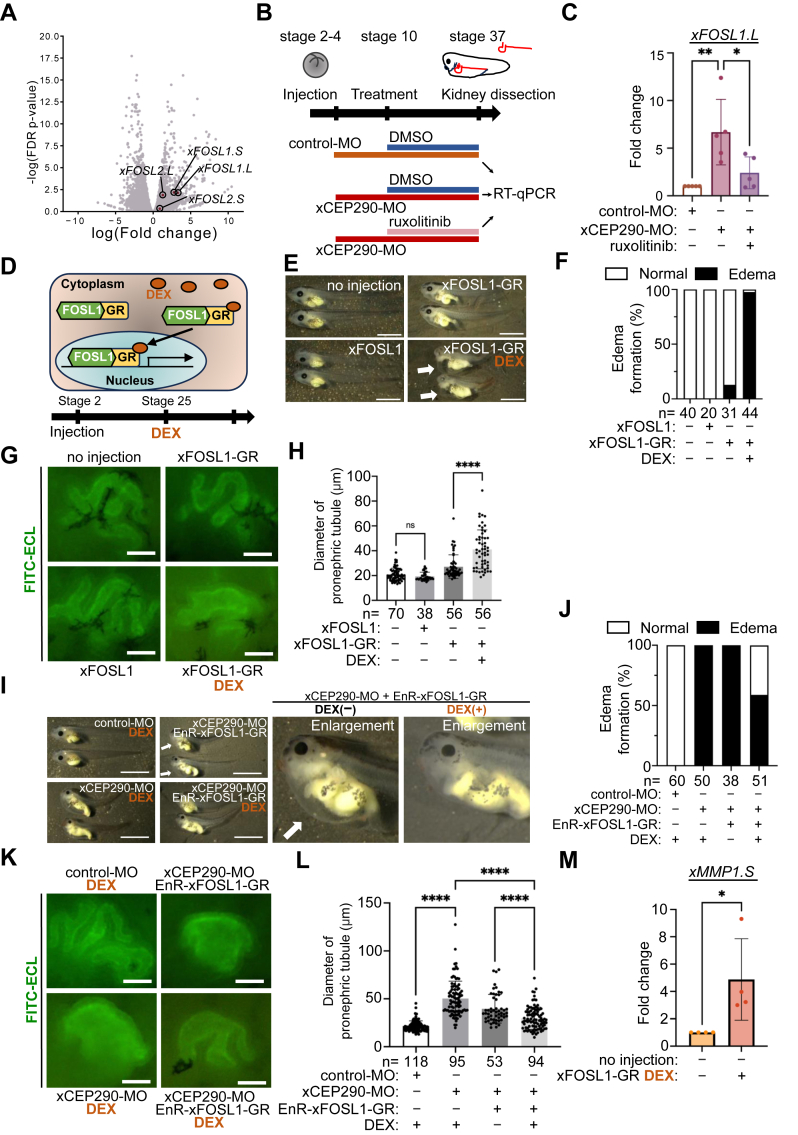
**Transcription factor FOSL1 induced renal phenotype of xCEP290 morphant**. *A*, Volcano plot between xCEP290-depleted kidneys and control kidneys in RNA-seq analysis. *xFOSL1* and *xFOSL2* genes are shown. *B*, experimental design of RT-qPCR. control-MO and xCEP290-MO (40 ng) were injected at stage 2 to 4; DMSO and ruxolitinib (1 μM) was treated from stage 10, followed by kidney dissection at stage 37. The dissected kidneys were pooled for RT-qPCR. *C*, *xFOSL1*.*L* gene expression was examined by RT-qPCR for each group in (*B*) from 5 independent experiments. ∗: *p* < 0.05, ∗∗: *p* < 0.01. *D*, experimental strategy with xFOSL1-GR protein. Upon binding with DEX, xFOSL1-GR protein translocates from cytoplasm to nucleus, and activates the transcription of xFOSL1 target genes. *E*, edema formation observed in xFOSL1-GR expressed *Xenopus* embryo with the DEX treatment. *Upper left panel*: no injection, *lower left panel*: wild type *xFOSL1* mRNA (500 pg), *upper right panel*: *xFOSL1-GR* mRNA (1 ng), and *lower right panel*: *xFOSL1-GR* mRNA (1 ng) with the DEX treatment (200 μM). Edemas are indicated by *white arrows* in the *lower right panel*. The scale bar represents 1 mm. *F*, the percentage of embryos with edema in examined embryos in (*E*). Each ‘n’ indicates the total number of embryos from three independent experiments. *G*, pronephric tubule was visualized using FITC-ECL staining (green). *Upper left panel*: no injection, lower left panel: *xFOSL1* mRNA (500 pg), *upper right panel*: *xFOSL1-GR* mRNA (1 ng), and *lower right panel*: *xFOSL1-GR* mRNA (1 ng) with the DEX treatment. The scale bar represents 50 μm. *H*, quantification of pronephric tubular inner diameter in examined embryos in (*G*). Each ‘n’ indicates the total number of embryos from three independent experiments. ∗∗∗∗: *p* < 0.0001. *I*, the observation of the edema in *EnR-xFOSL1-GR* mRNA co-injected xCEP290 morphant with or without the DEX treatment. *Upper left panel*: control-MO with the DEX treatment, *lower left panel*: xCEP290-MO with the DEX treatment, *upper middle panel*: xCEP290-MO with *EnR-xFOSL1-GR* mRNA (100 pg), and *lower middle panel*: xCEP290-MO with *EnR-xFOSL1-GR* mRNA (100 pg) and the DEX treatment. Enlarged images from the *upper* and *lower middle panels* are also shown. Edemas are indicated by *white arrows*. The scale bar represents 1 mm. *J*, the percentage of embryos with edema in examined embryos in (*I*). Each ‘n’ indicates the total number of embryos from three independent experiments. *K*, pronephric tubule was visualized by FITC-ECL staining (*green*). *Upper left panel*: control-MO with the DEX treatment, *lower left panel*: xCEP290-MO with the DEX treatment, *upper right panel*: xCEP290-MO with *EnR-xFOSL1-GR* mRNA (100 pg), and *lower right panel*: xCEP290-MO with *EnR-xFOSL1-GR* mRNA (100 pg) with the DEX treatment. The scale bar represents 50 μm. *L*, quantification of pronephric tubular inner diameter of in examined embryos in (*K*). Each ‘n’ indicates the total number of embryos from three independent experiments. ∗∗∗∗ indicates *p* < 0.0001. *M*, the expression level of *xMMP1* gene induced by overexpression of xFOSL1 in kidney by RT-qPCR. Expressions of genes were normalized with *xODC1* gene expression, and expressions in control groups were set as 1. Each data point represents the mean ± STDEV of three independent experiments were performed. ∗: *p* < 0.05.

The FOS family genes *FOSL1* and *FOSL2* are important transcription factors functional in inflammation and cell proliferation downstream of IL-6/JAK/STAT3 signaling (82). Since we observed the expression of *xFOSL1* was dynamically changed by xCEP290 knockdown and the treatment of tolvaptan as compared with *xFOSL2* (Figs. 3*E*, 5*A*), we first focused in examining a potential role of xFOSL1 for the formation of pronephric tubular dilation in xCEP290 morphant. We examined whether the expression of *xFOSL1* was quantitatively regulated by IL-6/JAK/STAT3 signaling in *Xenopus* embryonic kidneys. The expression of *xFOSL1* in xCEP290 morphant kidney was indeed up-regulated, and this up-regulation was suppressed by the ruxolitinib treatment (Fig. 5, *B* and *C*), indicating that *xFOSL1* gene expression was controlled by IL-6/JAK/STAT3 signaling in *Xenopus* embryonic kidney. To uncover the function of xFOSL1 in the formation of dilated pronephric tubule induced by xCEP290 knockdown, xFOSL1 was overexpressed in *Xenopus* embryos and embryonic morphologies were examined. No obvious morphological change was found in these embryos (Fig. 5*E*: lower left panel). This may be because overexpressed xFOSL1 protein may not persist into later stages when kidney development occurs because overexpressed xFOSL1 protein was degraded from the early stage (Fig. S6*A*). Therefore, we took advantage of hormone-inducible glucocorticoid receptor (GR) fusion protein, which is activated by exogenous application of dexamethasone (DEX) when needed and is also stable until activated (83, 84). GR-fused xFOSL1 (xFOSL1-GR) protein was overexpressed and stimulated by adding DEX at stage 25 (Fig. 5*D*), and indeed edema was induced by the overexpression of xFOSL1-GR in *Xenopus* embryo (Fig. 5*E*: lower right panel, F). While the length of primary cilia on the epithelial cells was not changed in xFOSL1-overexpressed kidney (Fig. S6, *B* and *C*), the diameter of pronephric tubule was also dilated in xFOSL1-GR overexpressed kidney (Fig. 5*G*: lower right panel, H, Fig. S6*D*). Next, we attempted to rescue morphological changes observed in xCEP290 morphant. When the suppressor domain of *Engrailed* gene (EnR) was fused to transcription factor, EnR-fused transcription factor, this construct can inhibit the transcription of downstream target genes (85). EnR and GR fused xFOSL1 (EnR-xFOSL1-GR) was generated, and EnR-xFOSL1-GR was overexpressed in xCEP290 morphant. Both edema (Fig. 5, *I* and *J*) and dilated pronephric tubule (Fig. 5, *K* and *L*, Fig. S6*D*) observed in xCEP290 morphant were improved by co-injection of *EnR-xFOSL1-GR* mRNA, implicating increased xFOSL1 expression in xCEP290 morphants can indeed produce the edema phenotype observed.

To explore the downstream genes of xFOSL1 in xCEP290 morphant kidney, we selected genes, whose expression was highly increased in xCEP290 morphant kidney compared with control-MO injected kidney and reduced by the treatment of tolvaptan (Data S-1), for further studies. In addition, some of these genes such as *xIL1B* and *xMMP1* have been reported as potential downstream of STAT3 (86, 87). The expression of these genes was examined in xFOSL1-overexpessed kidney, and we observed expression of xMMP1 was increased in xFOSL1-overexpressed kidney (Fig. 5*M*). Since the expression of other examined genes were not up-regulated by overexpression of xFOSL1 (Fig. S6*E*), the efficiency of rescue experiment for dilated pronephric tubules in xCEP290 morphant kidney by the inhibition of FOSL1 function is expected to be lower than the treatment of ruxolitinib (Fig. 4, *D* and *E*, Fig. 5, *K* and *L*).

These data indicate that xFOSL1 is a key transcription factor downstream of IL-6/JAK/STAT3 signaling in the formation of dilated pronephric tubule observed in xCEP290-depleted embryo.

To support the function of FOSL1 in cyst formation of human kidney, the expression of *FOSL1* and *FOSL2* genes were explored in human fetal kidney. UMAP plot of single-cell RNA-seq data using human developing nephron dataset, which were sourced from BJ Stewart *et al*. (88), showed clear segregation of various cell-types (Fig. S7*A*), with typical kidney cell markers distinctly localized within each cluster (Fig. S7*B*). The expression of *FOSL1* is predominantly expressed in distal and collecting duct cells (Fig. S7, *C* and *D*), and *FOSL2* expression was similar trend but was observed in a wider range of cells, such as podocyte, proximal tubular, and distal collecting cells (Fig. S7, *E* and *F*).

## Discussion

CEP290 is a pivotal protein for ciliary function, with mutations linked to a variety of ciliopathic symptoms including brain malformations, retinal degeneration and renal cyst formation. In this work, we have demonstrated that xCEP290-depleted embryos exhibited edema and dilated pronephric tubule with elongated cilia; the pronephric tubule, but not elongated cilia, was rescued by the treatment of tolvaptan, which is a vasopressin V_2_ receptor antagonist and prescribed for patients with ADPKD (68, 69, 71). RNA-seq analysis with xCEP290-depleted kidney indicated that IL-6/JAK/STAT3 signaling pathway is involved in the formation of pronephric tubular dilation in *Xenopu*s embryo. Furthermore, we show the transcription factor xFOSL1 plays an important role in the formation of pronephric tubular dilation downstream of IL-6/JAK/STAT3 signaling pathway in xCEP290 morphant. Our study discovered a key signaling axis in the formation of pronephric tubular dilation caused by abnormal cilia in xCEP290-depleted kidney.

The *CEP290* gene has been identified as a causative gene for JSRD (16, 19, 89, 90, 91) as it has been reported that patients with JSRD having *CEP290* gene mutations frequently suffer from kidney dysfunction (18, 19, 89, 92, 93). In this study, we examined the potential role of xCEP290 in dilated pronephric tubule formation using knockdown approaches in the *Xenopus* embryo. We observed that the length of cilia on the epithelial cells of the pronephric tubule in the xCEP290-depleted embryonic kidney was longer than those in wild-type embryos, which is consistent with the elongated cilia observed in fibroblasts from JSRD patients with *CEP290* gene mutations (17, 20). In addition, dilated pronephric tubules in the xCEP290-depleted embryo appear similar to the formation of cysts in the renal medulla of JSRD patients (94, 95), which is one of the characteristics of NPH in JSRD patients. Based on these findings, the abnormalities observed in xCEP290-depleted kidneys are thought to reproduce renal pathology of JSRD patients with *CEP290* gene mutations, and therefore, xCEP290-depleted embryos can be a useful animal model for studying the molecular mechanism of cyst formation in the kidneys of JSRD patients with *CEP290* gene mutations.

At present, no effective treatment has been established for NPH accompanied by patients having JSRD (15, 96). Progressive multiple cysts are observed in the kidney of ADPKD, a genetic disease classified as a ciliopathy (97, 98), and tolvaptan is used to slow the progression of cyst formation in ADPKD patients (71, 99). Although the causative genes of ADPKD, *PKD1* and *PKD2* genes encoding polycystin1 (PC1) and polycystin2 (PC2), respectively (100, 101), are different from those of JSRD, multiple cysts are also observed in NPH of JSRD patients, indicating that tolvaptan could be used for the treatment of NPH. This study demonstrated that the dilated pronephric tubule in xCEP290-depleted embryos was improved by the tolvaptan treatment. On the other hand, the structure of the pronephric tubule in the xCEP290-depleted embryo was not restored to the normal state of pronephric tubular extension by the tolvaptan treatment. These results suggest that CEP290 is involved not only in the formation of dilated pronephric tubules but also in the morphogenesis of the pronephric tubular structure, and that the signal(s) which regulates pronephric morphogenesis may not be related to the intracellular signal activated by vasopressin. While several signaling pathways were modulated in xCEP290-depleted conditions compared to control in the GSEA result of our RNA-seq analysis, not all signaling pathways, such as the MYC signal, were rescued by the tolvaptan treatment (Fig. S4, *E* and *F*). Since the tolvaptan treatment did not restore the elongated cilia phenotype, it is likely that not all abnormally activated ciliary signal pathways is blocked by the tolvaptan treatment. This result also supports our hypothesis that other signaling pathways, such as the MYC signal, are involved in the abnormal morphogenesis of the pronephric tubular extension observed in xCEP290-depleted embryos. Taken together, since the tolvaptan treatment recovered the formation of pronephric tubular dilation in xCEP290-depleted embryos, tolvaptan may be an effective therapeutic in the progression of NPH associated with JSRD.

While it has been reported that the IL-6/JAK/STAT3 signaling pathway is involved in the formation of multiple cysts observed in patients with ADPKD (102, 103, 104) not clear if this pathway is also involved in the pathogenesis of NPH associated with JSRD. In this study, we found the expression level of *IL-6* gene was increased in xCEP290-depleted kidneys and decreased by the tolvaptan treatment. We further showed that ruxolitinib, an inhibitor of JAK1/2 (77, 78, 105), improved the dilated pronephric tubule phenotype as well as activated xSTAT3 in the tubular epithelial cells observed in xCEP290-depleted kidney. Furthermore, the elongated cilia on the epithelial cells of pronephric tubules were not improved by ruxolitinib treatment in xCEP290 morphant kidneys, indicating that the IL-6/JAK/STAT3 signaling pathway is directly or indirectly activated by these elongated cilia which may then promote the dilation of pronephric tubule. IL-6 has been reported to exhibit extremely diverse biological activities and to play important roles in biological and pathological processes such as inflammation, immune response, and hematopoiesis (26, 106). However, how IL-6 signaling is involved in cyst formation of NPH as well as other cystic diseases has not been elucidated thus far. To explore the molecular mechanism of cyst formation induced by the abnormal activity of IL-6/JAK/STAT3 signaling pathway, we focused on *FOSL1*, a target gene of the IL-6/JAK/STAT3 signaling pathway (48, 49), whose gene expression was greatly increased in xCEP290 morphant kidney in our RNA-seq analysis. While FOSL1 is known to play an important role in various biological processes (52, 53, 107), its involvement in cyst formation has not been reported. We here showed that overexpression of xFOSL1 could induce the formation of dilated pronephric tubule in embryonic kidney, and overexpression of xFOSL1 fused to the repressor domain of *Engrailed* gene rescued dilated pronephric tubule observed in xCEP290-depleted kidney. In addition, the high expression level of *xFOSL1* gene in xCEP290 morphant kidney was restored to normal level by the ruxolitinib treatment. This is the first study that reveals that abnormal expression of xFOSL1 caused the formation of dilated pronephric tubule, resembling renal cyst observed in JSRD, downstream of IL-6/JAK/STAT3 signaling. While we demonstrate the expression of *xMMP1* gene is regulated by xFOSL1, how xFOSL1 induces the formation of dilated pronephric tubule remains to be determined in future studies.

The expression pattern of *FOSL1* in human kidney supports that the signaling pathways involving FOSL1 are capable of functioning in the renal tubules. Therefore, our results strongly indicate that the IL-6/JAK/STAT3/FOSL1 signal axis is crucial for the pathogenesis of NPH associated with JSRD, but how ciliary abnormalities caused by CEP290 dysfunction induce abnormal expression levels of *IL-6* remains unknown. Our study also demonstrated the potential of ruxolitinib in the treatment of NPH in patients with JSRD and suggested FOSL1 as a new therapeutic target. These results need to be verified in mammalian experimental systems, including organoids derived from human cells such as iPS cells originating from JSRD patients, but these drugs and target molecules have provided new resources for developing a treatment for NPH associated with JSRD.

## Experimental procedures

### *X*. *laevis* embryo manipulations

Eggs were artificially fertilized with testis homogenates and cultivated in 20% Steinberg’s solution (58 mM NaCl, 0.68 mM KCI, 0.34 mM Ca(NO_3_)_2_ 4H_2_O, 0.83 mM MgSO_4_ 7H_2_O, 10 mM Herpes; pH 7.4 to 7.6) (108). Embryos were staged according to Nieuwkoop and Faber (109). The studies using *Xenopus* were approved by the review boards of Nagoya City University.

### DNA constructs

*xFOSL1* was subcloned into site between StuI and XhoI of pCS2+ vector. For 3Flag-xFOSL1-GR (pCS2+-3Flag-xFOSL1-GR), *xFOSL1* was subcloned into site between StuI and XhoI of pCS2+-3Flag-GR vector. For 3Flag-xFOSL1, the GR domain of 3Flag-xFOSL1-GR was removed. For EnR-xFOSL1-GR (pCS2+-EnR-xFOSL1-GR), *xFOSL1* was subcloned into site between StuI and XhoI of pCS2+-EnR-GR vector. For xCEP290(-52∼394)-2HA (pCS2+-xCEP290(-52∼394)-2HA) containing morpholino oligonucleotide (MO) site of *xCEP290*, *xCEP290* (−52∼394) was subcloned into site between EcoRI and XhoI of pCS2+-2HA using In-Fusion HD Cloning Kit (Takara, 639,650). Primers used for subcloning were shown in Table S1.

### Synthetic mRNA, morpholino oligonucleotides and microinjection

Capped synthetic mRNAs were generated by *in vitro* transcription with SP6 RNA polymerase, using the mMESSAGE mMACHINE SP6 Transcription kit (Thermo Fisher Scientific, AM1340). Antisense MOs and standard control were purchased from Gene Tools. The MOs used were control-MO (CCTCTTACCTCAGTTACAATTTATA) which has no target but very little activity that corrects a splicing error by a mutation at position 705 of beta-globin pre-mRNA in reticulocytes from thalassemic humans, and xCEP290-MO (ATCCAGAGAAGGCGGCATCTTTAAG). For microinjections, 2- or 4-cell stage embryos were placed in 3% Ficoll in 50% Steinberg’s solution, injected with 10 nl or 20 nl of the specified amount of mRNA solutions and cultured in 20% Steinberg’s solution until the desired stage. membrane RFP (mRFP) RNA was co-injected as a tracer.

### Treatment with small molecules

All compounds were dissolved in DMSO to make stock solutions as follows. Tolvaptan (50 mM, Sigma-Aldrich, T7455), ruxolitinib (10 mM, MedChemExpress, HY-50856), and dexamethasone (20 mM, Nacalai Tesque, 11,107–64). The stock solutions were diluted for treating concentrations in 20% Steinberg’s solution to prepare treatment solution. For the small molecule treatment, embryos were transferred into the treatment solutions. Embryos treated with equivalent DMSO concentrations without the small molecules were shown as DMSO treatment.

### Protein extract and immunoblot analyses

Pooled *Xenopus* 10 embryos at stage 10 were homogenized with 0.5% Triton-X lysate buffer containing 20 mM Tris-HCl (pH 8.0), 5 mM MgCl_2_, 1 mM EDTA, 50 mM KCl, 0.5% Triton X-100, 10% glycerol, 1 mM DTT, and protease inhibitors (2 μg/ml aprotinin, 10 μ g/ml leupeptin, 1 mM PMSF, 20 μg/ml trypsin inhibitor). Extracted proteins were separated by 10 or 15% SDS-PAGE followed by transfer from the gel onto membranes (Bio-Rad). The membranes were incubated with 1 h in phosphate buffer saline (PBS) containing 0.05% Tween 20 and 5% skim milk, and incubated overnight with anti-Flag (Sigma-Aldrich, F3165, 1/10,000), anti-HA (Roche, 11,583,816,001, 1/1000), or anti-GAPDH (Novus Biologicals, NF300–322, one-fifth000) antibodies. Secondary anti-mouse IgG-HRP for anti-HA antibody and anti-rabbit IgG-HRP for anti-GAPDH antibodies were used. Gel images were obtained by Amersham Imager (GE Healthcare).

### *Erythrina crist-agalli* lectin staining

Embryos were fixed at stage 37 in 3-(N-morpholino) propanesulfonic acid/EGTA/magnesium sulfate/formaldehyde (MEMFA) fixative (0.1 M MOPS, 2 mM EGTA (pH 8.0), 1 mM MgSO_4_, 3.7% formaldehyde) at 4 °C overnight (110) and washed and kept in PBS at 4 °C. Head and tail part were removed to dissect trunk part of embryo. The dissected embryos were treated with methanol for 20 min at room temperature and washed in PBS. After treating with 0.1% Triton X-100 and 0.2% BSA in PBS (PBT), the embryos were incubated with 10% goat serum in PBT at room temperature for 1 h, followed by incubation with FITC-conjugated *E*. *crist-agall*i lectin (FITC-ECL: Vector Laboratories, FL-1141, 1/100) at 4 °C overnight. After washing with PBT, embryos were stored in PBS. After removing epidermis, kidney images were taken by epifluorescence microscope (Olympus, SZX7, Japan) equipped with digital camera (Advan Cam-E3Rs, AdvanVision).

### Fluorescence immunohistochemistry

Embryos were fixed in MEMFA fixative at 4 °C overnight, washed in PBS at 4 °C and incubated in 30% sucrose (Nacalai, 09,589–05) in PBS for 1 day at 4 °C. Embryos were embedded in OCT compound (Sakura Fintek, 4583) at −30 °C, and frozen sections were cut at a thickness of 16 μm. Slides were washed and stored in PBS at 4 °C. After washing in PBT, the slides were incubated with 10% goat serum in PBT at room temperature for at least 1 h, followed by incubation with anti-acetylated-α-tubulin mouse IgG antibody (Sigma-Aldrich, T6793, 1/500) and FITC-ECL (Vector Laboratories, FL-1141, 1/100) at 4 °C overnight. The primary antibodies were detected with Alexa Flour 594 goat anti-mouse IgG antibody (Thermo Fisher Scientific, A11005, 1/500), and nucleus were counterstained with DAPI (Sigma-Aldrich, D9542, 4 μg/ml), respectively. The stained slides were mounted with Flouromount-G (Southern Biotech, 0100–01).

### Whole mount fluorescence immunohistochemistry

Embryos were fixed at stage 37 in MEMFA fixative at 4 ^o^C overnight, followed by thorough washes in PBS. The head and tail regions were removed to dissect trunk part of embryos. The dissected embryos were treated with methanol for 20 min at room temperature, and subsequently washed in PBS. Following three washes in PBT, embryos were incubated with 10% goat serum in PBT at room temperature for 1 h. Then incubated at 4 ^o^C overnight with the following primary antibodies: mouse acetylated-*α*-tubulin antibody (Sigma-Aldrich, T6793, 1/100), FITC-ECL (Vector Laboratories, FL-1141, 1/100) and rabbit anti-phosphorylated-xSTAT3 (p-STAT3: Tyr705) (Cell Signaling, 9145, 1/200). The primary antibodies were detected with Alexa Flour 594 goat anti-mouse IgG secondary antibody (Thermo Fisher Scientific, A11005, 1/500), Alexa Flour 594 goat anti-rabbit IgG secondary antibody (Thermo Fisher Scientific, A11037, 1/500), and nucleus were counterstained with DAPI (Sigma-Aldrich, D9542, 4 μg/ml), respectively. After additional PBT washes, embryos were stored in PBS until imaging.

To facilitate imaging cilia on the epithelial cells of pronephric tubules, the epidermis overlying the pronephric tubule region was carefully removed. Cilia length on apical surface of epithelial cells lining pronephric tubules was manually measured using Fiji software (version:2.14.0/1.54) from confocal z-stack images acquired with confocal microscope (Olympus, FV3000).

For analysis of p-STAT3 expression, the epidermis overlying the pronephric tubule region was carefully removed, and then images were obtained using a confocal microscopy (Olympus, FV3000).

### Image analysis

Left and right sides of kidney images were captured and analyzed using Fiji software (version:2.14.0/1.54), where the proximal tubular region of kidney was delineated with FITC-ECL signal. Measurements of pronephric tubular inner diameter were randomly obtained at three distinct anatomical locations within each kidney, and the mean diameter was calculated to ensure accuracy. For the measurement of cilia length on the epithelial cells of the pronephric tubules, images were obtained using a confocal microscopy (Olympus, FV3000), and the cilia lengths were manually measured by Fiji software (version:2.14.0/1.54).

### Kidney dissection and isolation of RNA

The kidneys were dissected from *Xenopus* embryos stage 37 on agar-coated dishes containing 1 × Steinberg’s solution, using a tungsten needle. Total RNA from dissected kidney was extracted using the RNeasy Micro Kit (Qiagen, 74004) and quality and concentration were initially determined using a Nanodrop spectrophotometer and RNA integrity measured using an Agilent 2100 Bioanalyzer G2939A to Library preparation. All samples had RNA integrity number equivalent values greater than 8.

### Quantitative RT-PCR

cDNA was synthesized by ReverTra Ace pPCR Master Mix with gDNA remover (TOYOBO, FSQ-301) according to the manufacturer’s instructions. RT-qPCR analysis was performed with Fast start Universal Master (Roche, 04913914001) using a QuantiStudio 12K Flex System (Thermo Fisher Scientific). All primers were used at a final concentration of 400 nM. Primer sequences used are given in Table S2. The comparative *C*_t_ method was used to determine the relative quantities of mRNA, using *xODC1* mRNA as the endogenous reporter. Each RNA sample was analyzed in duplicate. Each data point represents the mean ± STDEV of at least three independent experiments.

### RNA-seq analysis

For RNA-seq analysis, RNAs were isolated from kidneys dissected form xCEP290 knockdown embryos with or without tolvaptan as well as control embryos at stage 37 with the RNeasy Macro Kit (Qiagen, 74004). Libraries were prepared from these extracted RNAs using KAPA Stranded mRNA-Seq Kit (KAPA Biosystems, KK8420), KAPA Universal Adapter (KAPA Biosystems, 9063781,001) and KAPA UDI Primer Mixes, 1 to 96 reactions (Roche, 09134336001) according to the manufacturer’s instructions. The resulting cDNA library products were sequenced using the NovaSeq 250 (Illumina) to obtain 2 × 150 bp paired end reads. The obtained raw reads were analyzed by CLC genomics workbench 20.0.4, a commercial software.

### Principal component analysis

Following exclusion of genes exhibiting read counts of 10 or fewer across all samples, the log-transformed TPM values plus 1 were standardized using the StandardScaler of the scikit-learn Python package. Principal component analysis (PCA) and calculation of the resulting explained variances were subsequently performed utilizing the same package.

### GSEA analysis

*X*. *laevis* Entrez gene IDs were retrieved from gene symbols using DAVID ID conversion tool, while human Entrez gene IDs and corresponding gene symbols were sourced from NIBI (txid = 9606). These datasets were integrated to generate a human and *X*. *laevis* ortholog gene dataset by merging the same gene symbol. Human Entrez gene IDs from the hallmark gene sets (h.all.v2023.1.Hs.entrez.gmt) were subsequently converted to *X*. *laevis* Entrez gene IDs. Gene set enrichment analysis (GSEA) was performed using the pre-ranked function of GSEApy Python package.

### Single-cell RNA-seq analysis of human kidney data

Human fetal kidney single-cell RNA-seq data were sourced from BJ Stewart *et al*. (88), with fetal developing nephron dataset was used for the analysis. Genes detected in three or fewer cells were filtered out, along with cells exhibiting fewer than 2000 expressed genes and those showing elevated mitochondrial gene expression. Gene expression levels were normalized to expression levels per 10,000 reads and log transformed. PCA was performed for dimensionality reduction, followed by the construction of a nearest neighbor distance matrix with parameters of n_neighbors = 40, n_pcs = 5. Based on this matrix, UMAP projection was generated using the scanpy Python package. The Leiden algorithm separated 8 clusters and finally consolidated into 4 clusters based on expression patterns of marker genes. Kidney cell-type specific marker genes were selected by referencing established literatures (111, 112).

### Statistics

Statistical analyses were performed using one-way ANOVA, followed by Tukey’s multiple comparison test. All statistical analyses were performed using GraphPad Prism Software (version 10.5.1). *p* values less than 0.05 indicate significant differences between groups.

## Data availability

RNA-seq data files have been deposited to the DDBJ Sequence Read Archive (DRA) with the dataset identifier PRJDB19584.

## Supporting information

This article contains supporting information.

## Conflict of interest

The authors declare that they have no conflicts of interest with the contents of this article.
