# Supplementary material for: Aberrant activation of IL-6/JAK/STAT3/FOSL1 signaling induces renal abnormalities in a Xenopus model of Joubert syndrome-related disorders
Source: J Biol Chem. 2025 Jun 24;301(8):110413. doi: 10.1016/j.jbc.2025.110413 (PMC12302719; doi:10.1016/j.jbc.2025.110413)
Supplement: Supplementary Data 1 [file mmc1.docx]

**Supporting information**

**Figures**

**
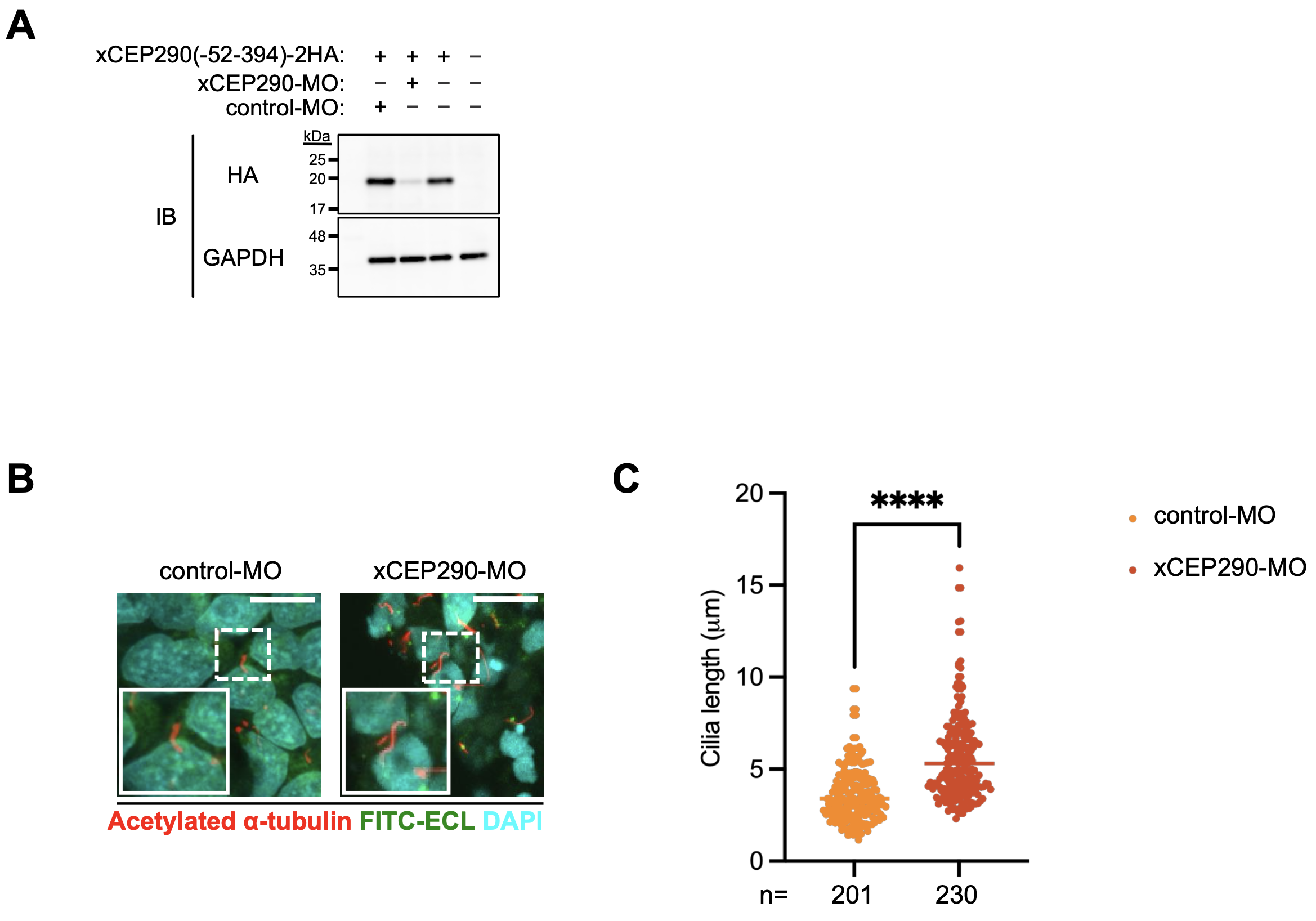
**

**Figure S1**

(A) The knockdown efficiency of xCEP290-MO in *Xenopus* embryo. *xCEP290(-53-394)-2HA* mRNA (500pg) encoding HA-tagged xCEP290 N-terminal was co-injected with control-MO (40ng) and xCEP290-MO (40ng). Expression of xCEP290(-53-394)-2HA protein at stage 10 was examined by immunoblot analysis. GAPDH was used as a loading control. (B) Immunofluorescence analysis of cilia in pronephric tubes at stage 37. Cilia, pronephric tubes, and nuclei were stained with anti-acetylated α-tubulin antibody (red), FITC-ECL (green), DAPI (cyan), respectively. Left panel: control-MO (40 ng), and right panel: xCEP290-MO (40 ng). A cilium surrounded by dotted lines is enlarged and shown in a lower left white square. Images were obtained using a confocal microscopy (Olympus, FV3000). The scale bar represents 10 µm. (C) Quantification of cilia length in pronephric tubules. Each ‘n’ indicates the total number of cilia from three independent experiments. ****: p<0.0001.

**
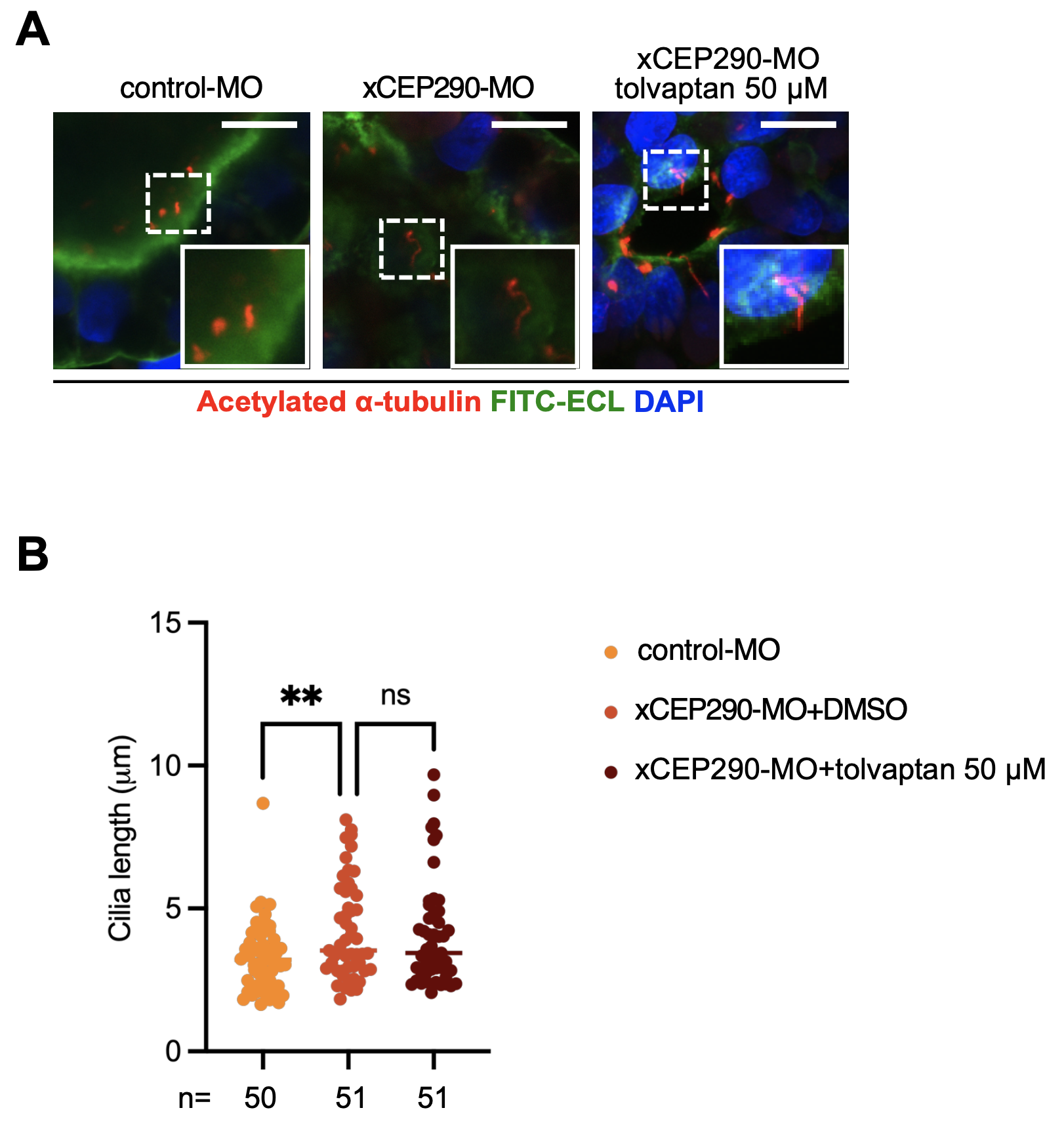
**

**Figure S2**

(A) Immunofluorescence analysis of cilia in pronephric tubes at stage 37. Cilia, pronephric tubes, and nuclei were stained with anti-acetylated α-tubulin antibody (red), FITC-ECL (green), DAPI (cyan), respectively. Left panel: control-MO (40 ng), middle panel: xCEP290-MO (40 ng), right panel: xCEP290-MO (40 ng) with tolvaptan treatment. A cilium surrounded by dotted lines is enlarged and shown in a lower right white square. Images were obtained using a confocal microscopy (Olympus, FV3000). The scale bar represents 10　µm. (B) Quantification of cilia length in pronephric tubules. Each ‘n’ indicates the total number of cilia from two independent experiments. *: p<0.05, ns: no significance.

**
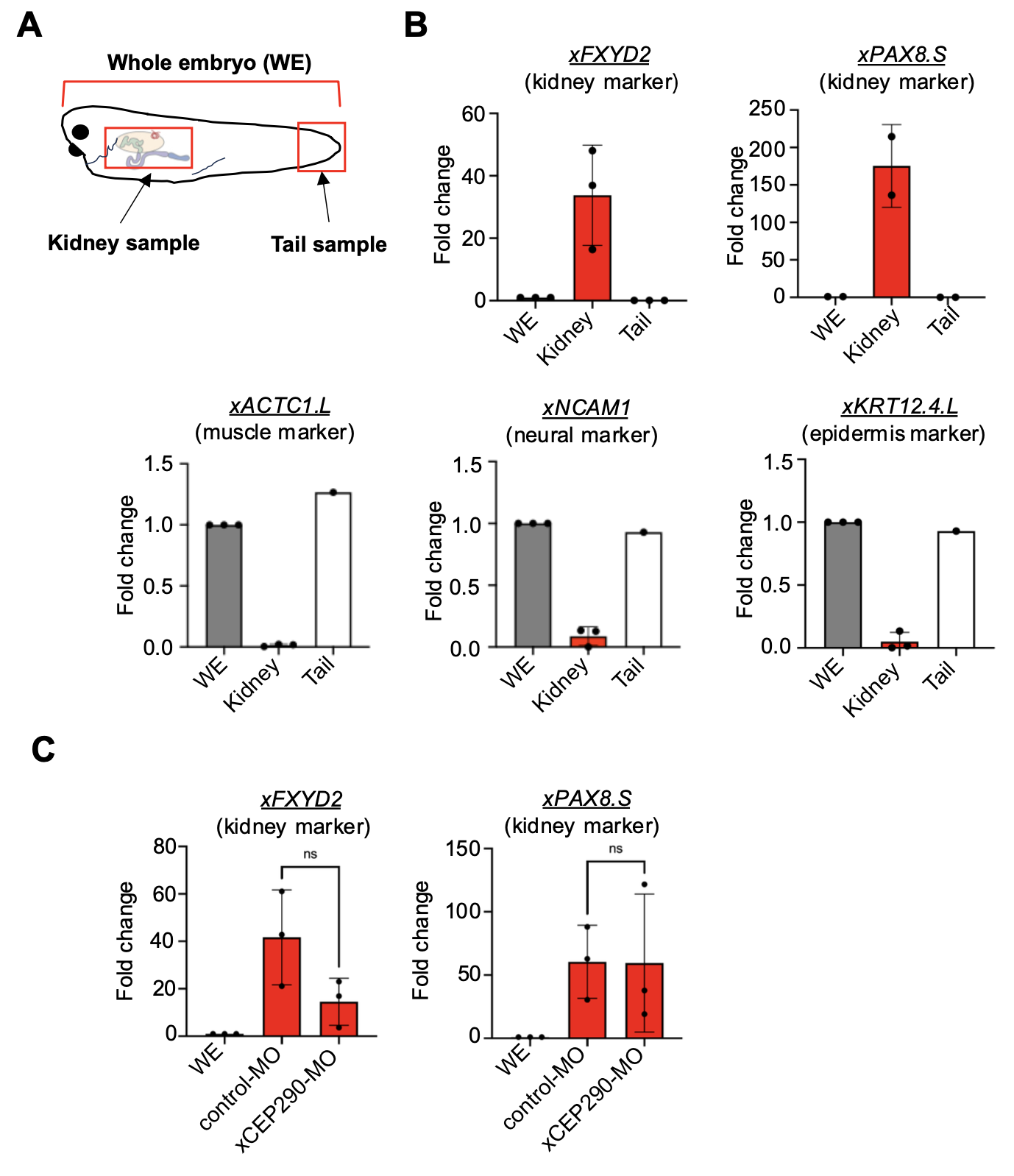
**

**Figure S3**

(A) Schematic showing dissected *Xenopus* tissues. The region of whole embryo (WE), kidney and tail are shown. (B) RT-qPCR analysis with *Xenopus* embryonic kidney. *xODC1* was used for normalization. Kidney markers: *xFXYD2* and *xPAX8*, muscle marker: *xACTC1*, neural marker: *xNCAM1*, and epidermis marker: *xKERT12.4*. (C) *xFXYD2* and *xPAX8* expression was examined by RT-qPCR in xCEP290 morphant kidney. ns: no significance.

**
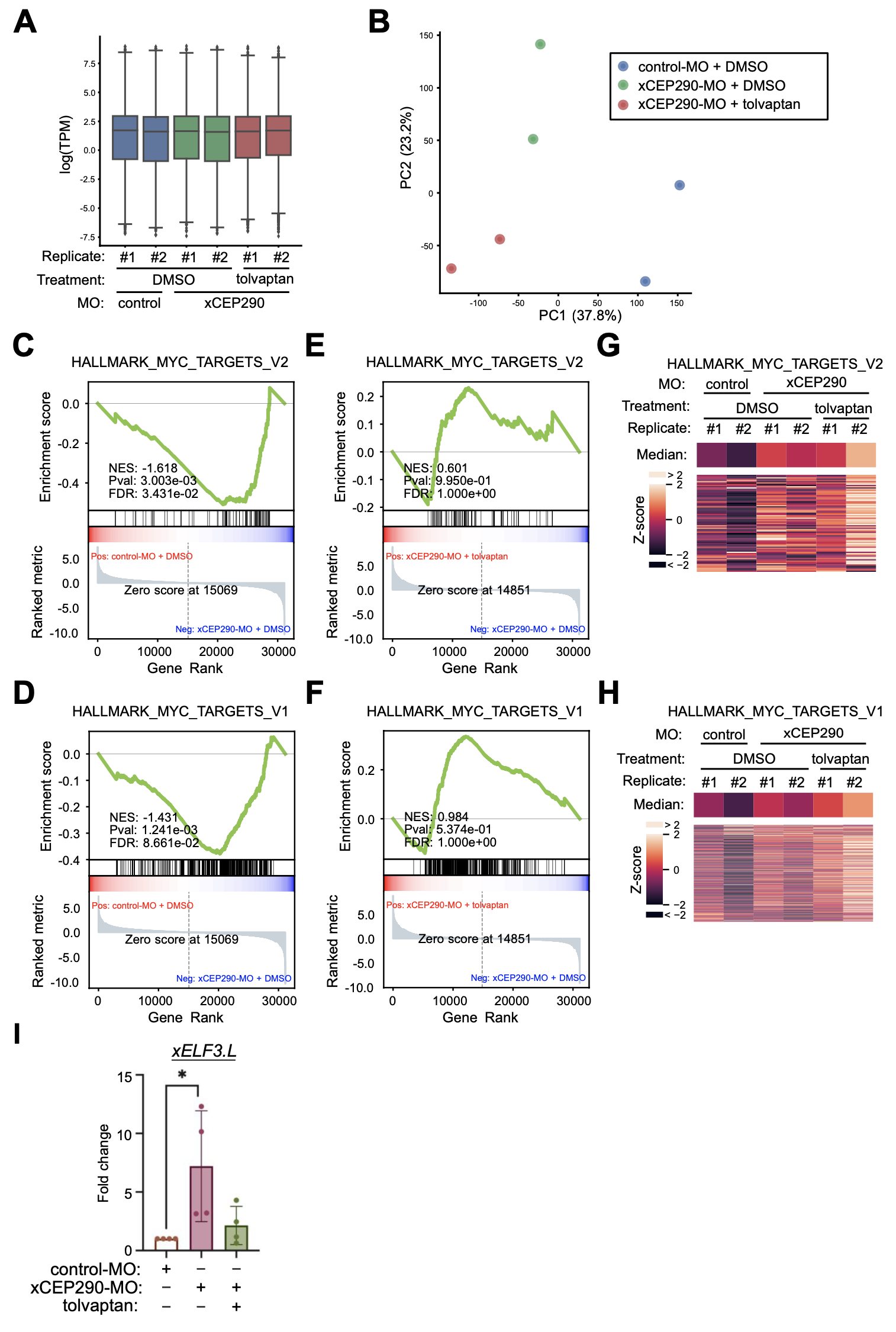
**

**Figure S4**

(A) Box plot of log-transformed TPM values between samples in *Xenopus* kidney RNA-seq analysis. (B) PCA plot of RNA-seq analysis with *Xenopus* kidney. Each color indicates the condition and treatment. (C) GSEA plot in HALLMARK_MYC_TARGETS_V2 between control-MO and xCEP290-MO in DMSO treatment condition. (D) GSEA plot in HALLMARK_MYC_TARGETS_V1 between control-MO and xCEP290-MO in DMSO treatment condition. (E) GSEA plot in HALLMARK_MYC_TARGETS_V2 between DMSO and tolvaptan treatments in xCEP290-MO condition. (F) GSEA plot in HALLMARK_MYC_TARGETS_V1 between DMSO and tolvaptan treatments in xCEP290-MO condition. (G) Heatmap plot of genes in the HALLMARK_MYC_TARGETS_V2 gene set. (H) Heatmap plot of genes in the HALLMARK_MYC_TARGETS_V1 gene set. (I) RT-qPCR validation analysis of *xELF3.L* gene expression in indicated conditions. *xODC1* was used for normalization, and expressions in control groups were set as 1. Each data point represents the mean ± STDEV of three independent experiments were performed. *: p<0.05

**
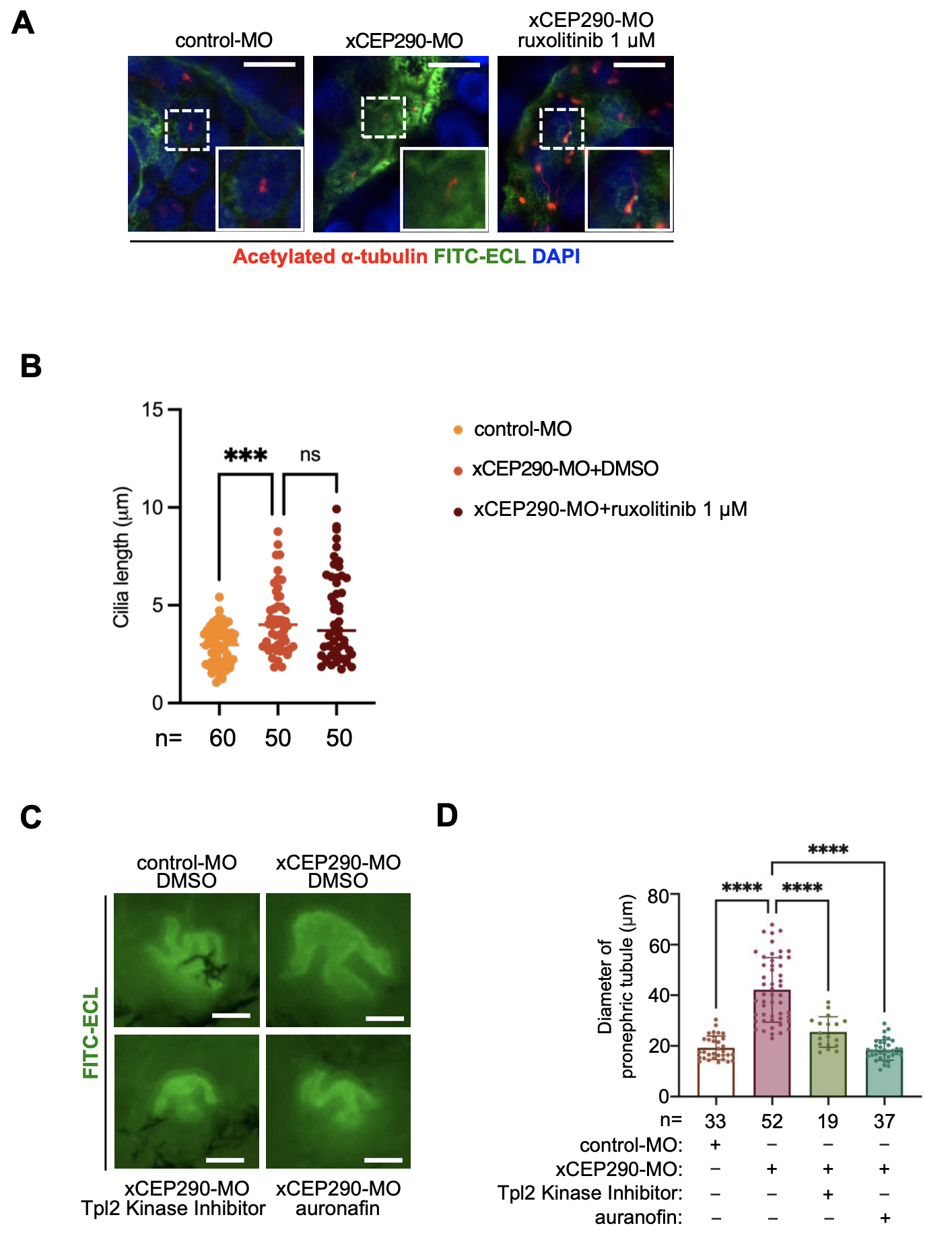
**

**Figure S5**

(A) Immunofluorescence analysis of cilia in pronephric tubes at stage 37. Cilia, pronephric tubes, and nuclei were stained with anti-acetylated α-tubulin antibody (red), FITC-ECL (green), DAPI (cyan), respectively. Left panel: control-MO (40 ng), middle panel: xCEP290-MO (40 ng), right panel: xCEP290-MO (40 ng) with ruxolitinib treatment. A cilium surrounded by dotted lines is enlarged and shown in a lower right white square. Images were obtained using a confocal microscopy (Olympus, FV3000). The scale bar represents 10 µm. (B) Quantification of cilia length in pronephric tubules. Each ‘n’ indicates the total number of cilia from two independent experiments. ***: p<0.001, ns: no significance. (C) Pronephric tubule was visualized using FITC-ECL staining (green). Upper left panel: control-MO with the DMSO treatment, upper right panel: xCEP290-MO with the DMSO treatment, lower left panel: xCEP290-MO with Tpl2 Kinase Inhibitor (FUJIFILM and Wako, 19710, 1 µM), and lower right panel: xCEP290-MO with auronafin (Cayman Chemical, 012-25081, 10 µM). Images were obtained using a confocal microscopy (Olympus, FV3000). The scale bar represents 50 µm. (D) Quantification of pronephric tubular inner diameter in examined embryos in (C). Each ‘n’ indicates the total number of embryos from three independent experiments. ****: p<0.0001.

**
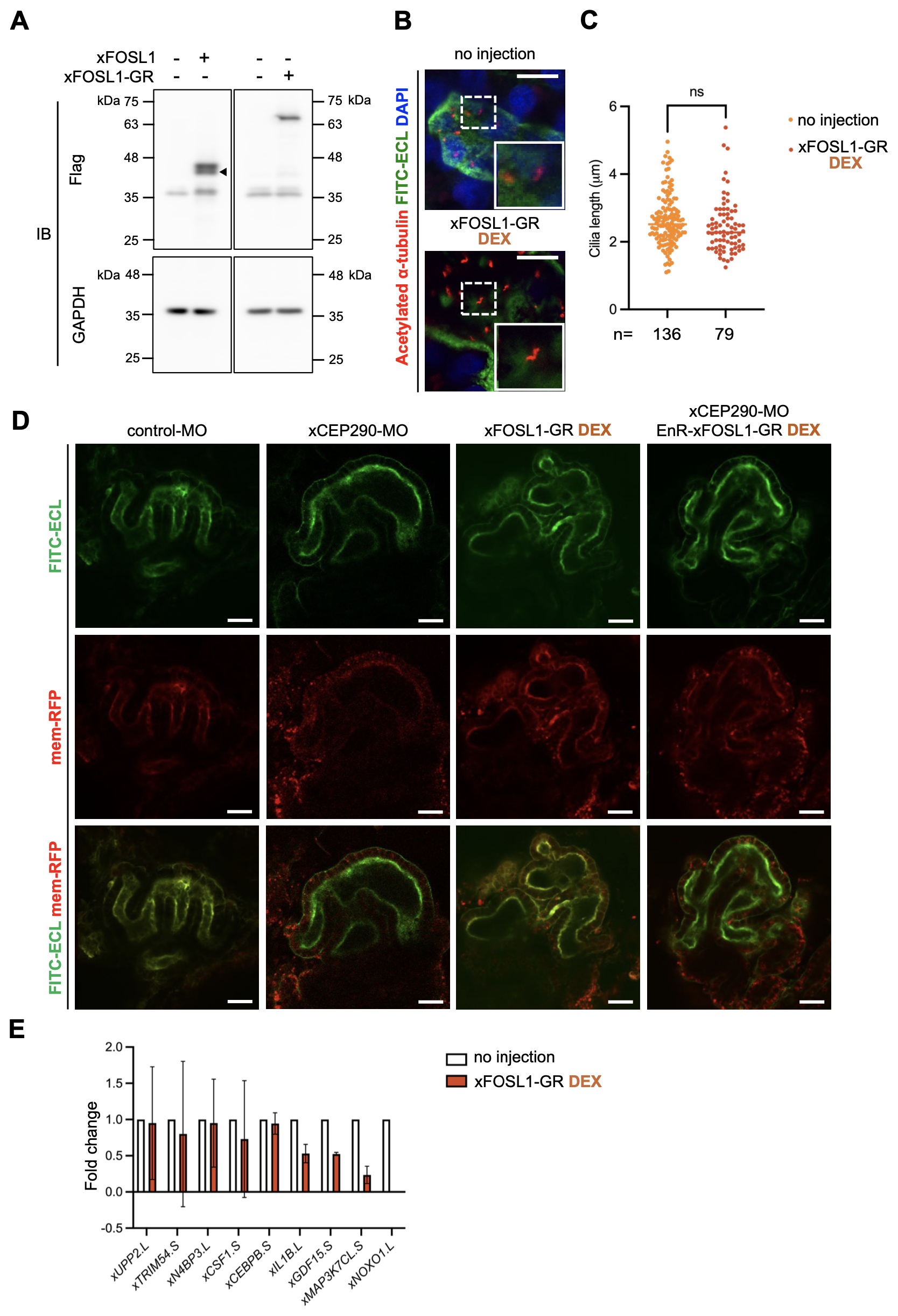
**

**Figure S6**

(A) The expression of 3Flag-xFOSL1 and 3Flag-xFOSL1-GR proteins. *3Flag-xFOSL1* or *3Flag-xFOSL1-GR* mRNA was injected, and each protein was examined at stage 10 by immunoblot analysis. GAPDH was used as a loading control. Degraded 3Flag-xFOSL1 is indicated by a black arrowhead. (B) Immunofluorescence analysis of cilia in pronephric tubes at stage 37. Cilia, pronephric tubes, and nuclei were stained with anti-acetylated α-tubulin antibody (red), FITC-ECL (green), DAPI (cyan), respectively. Upper panel: no injection, and lower panel: xFOSL1-GR with DEX treatment. A cilium surrounded by dotted lines is enlarged and shown in a lower right white square. Images were obtained using a confocal microscopy (Olympus, FV3000). The scale bar represents 10 µm. (C) Quantification of cilia length in pronephric tubules. Each ‘n’ indicates the total number of cilia from two independent experiments. ns: no significance. (D) control-MO, xCEP290-MO, xFOSL1-GR RNA and EnR-xFOSL1-GR RNA were injected in pronephric tubular epithelial cells of *Xenopus* embryonic kidney. Epithelial cells of pronephric tubule were visualized using FITC-ECL staining (green). mRFP RNA (red) was co-injected as a tracer of injection. Images were obtained using a confocal microscopy (Olympus, FV3000). The scale bar represents 40 µm. (E) The expression level of genes induced by overexpression of xFOSL1 in kidney by RT-qPCR. Expressions of genes were normalized with xODC1 gene expression, and expressions in control groups were set as 1. Each data point represents the mean ± STDEV of two independent experiments were performed.

**
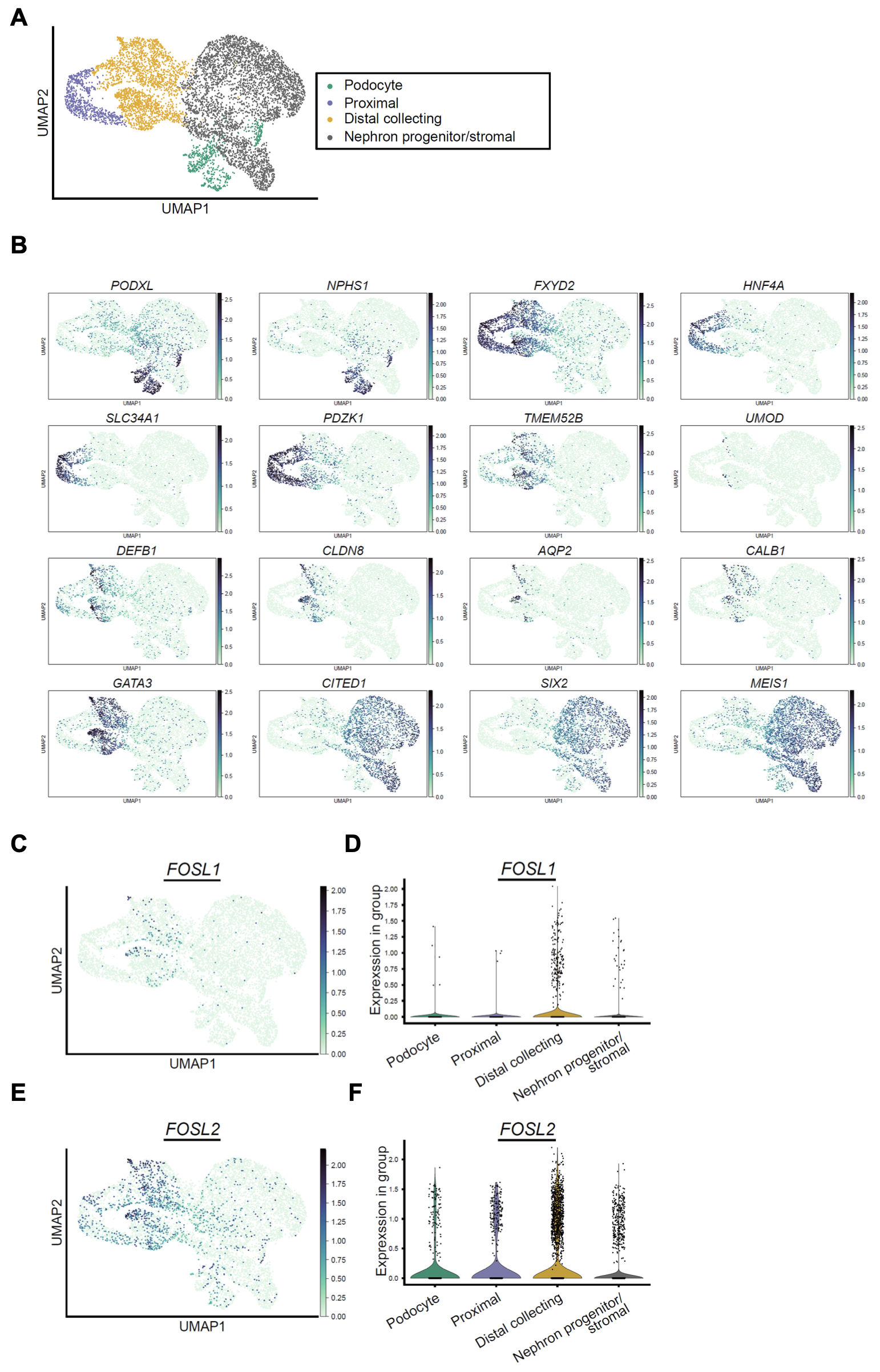
**

**Figure S-7**

(A) UMAP plot of human fetal kidney single-cell RNA-seq data. Different cell-types in fetal kidney cells were represented in different colors. (B) UMAP plot of typical kidney cell markers (1, 2). podocyte marker: *PODXL* and *NPHS1*, tubular cell marker: *FXYD2* and *HNF4A*, proximal tubule markers: *SLC34A1* and *PDZK1*, distal tubule marker: *TMEM52B* and *UMOD*, distal tubule/collecting duct marker: *DEFB1*, collecting duct marker: *CLDN8* and *AQP2*, ureteric bud/collecting duct marker: *CALB1* and *GATA3*, nephron progenitor marker: *CITED1* and *SIX2*, and stromal marker: *MEIS1*. (C) *FOSL1* expression pattern in the UMAP of (A) is shown. Color bar indicates the log-transformed normalized expression. (D) Violin plot of the *FOSL1* expression between cell-types. Dot plot of each single cell is also shown. (E) *FOSL2* expression pattern in the UMAP of (A) is shown. Color bar indicates the log-transformed normalized expression. (F) Violin plot of the *FOSL2* expression between cell-types. Dot plot of each single cell is also shown.

**References**

1. Bais, A. S., Cerqueira, D. M., Clugston, A., Bodnar, A. J., Ho, J., and Kostka, D. (2021) Single-cell RNA sequencing reveals differential cell cycle activity in key cell populations during nephrogenesis. *Scientific Reports 2021 11:1*. **11**, 1–15
2. Liao, J., Yu, Z., Chen, Y., Bao, M., Zou, C., Zhang, H., Liu, D., Li, T., Zhang, Q., Li, J., Cheng, J., and Mo, Z. (2020) Single-cell RNA sequencing of human kidney. *Scientific Data 2020 7:1*. **7**, 1–9

**Data**

**Data S-1:** RNA-seq results with CEP290 morphant kidney of *Xenopus* embryos

**Tables**

**Table S-1:** Primers used for subcloning

**Table S-2:** Primer used for RT-qPCR
